# Patient- and Prescriber-Related Factors Associated with Potentially Inappropriate Medications and Drug–Drug Interactions in Older Adults

**DOI:** 10.3390/jcm10112305

**Published:** 2021-05-25

**Authors:** Suhyun Jang, Sohyun Jeong, Sunmee Jang

**Affiliations:** 1 Gachon Institute of Pharmaceutical Sciences, College of Pharmacy, Gachon University, Incheon 21936, Korea; shjang@gachon.ac.kr; 2 Marcus Institute for Aging Research, Hebrew Senior Life, Boston, MA 02131, USA; twink9114@hanmail.net; 3 Department of Medicine, Beth Israel Deaconess Medical Center, Harvard Medical School, Boston, MA 02446, USA

**Keywords:** observational study, Beers Criteria, potentially inappropriate medication, drug–drug interaction, older adults, continuity of care

## Abstract

We aimed to evaluate the prevalence of potentially inappropriate medication (PIM) use and drug–drug interactions (DDIs) in older adults and their associated factors. This cross-sectional study used National Health Insurance data of older adults in South Korea. The 2015 AGS Beers Criteria were used to classify PIM use and DDIs. The associations of PIM use and DDIs with patient- and prescriber-related factors were evaluated using multiple logistic regression. Of the older adults who received at least one outpatient prescription (*N* = 1,277,289), 73.0% and 13.3% received one or more prescriptions associated with PIM use or DDIs, respectively. Chlorphenamine was most commonly associated with PIM, followed by diazepam. Co-prescriptions of corticosteroids and NSAIDs accounted for 82.8% of DDIs. Polypharmacy and mainly visiting surgeons or neurologists/psychiatrists were associated with a higher likelihood of prescriptions associated with PIM use or DDIs. Older age, high continuity of care (COC), and mainly visiting a hospital were associated with a lower likelihood of PIM use or DDIs. Prescriptions associated with PIM use and DDIS were more frequent for low COC patients or those who mainly visited clinics; therefore, patients with these characteristics are preferred intervention targets for reducing prescriptions associated with PIM use and DDIs.

## 1. Introduction

With the global population aging and the concomitant increase in chronic diseases, older populations may have an increased likelihood of multimorbidity [1]. Drug therapy is the most relevant therapeutic intervention in medicinal care; thus, older adults with multimorbidity are likely to receive multiple drug treatments (polypharmacy [2]). Polypharmacy is defined differently depending on the study, but several studies have defined it as taking more than five drugs simultaneously [3]. Polypharmacy may increase the complexity of the dosing regimen, which may be problematic in patients with cognitive problems. This increases the risk of the prescription of potentially inappropriate medications (PIMs). PIM use in older adults causes adverse drug reactions, which may result in falls, fractures, hospitalization, and death; furthermore, it also complicates the treatment regimen and increases health care costs [4,5,6].

The Beers Criteria, the most commonly used system, are explicit criteria to improve the selection of prescription drugs and to facilitate appropriate use of drugs; thus, the Beers Criteria are used for evaluating the quality of care and the patterns of drug use among older adults [7]. Two components have been added to the 2015 Beers Criteria: a list of drug–drug interactions (DDIs) and drugs for which dose adjustment is required according to kidney function. South Korea (hereafter, Korea) has experienced faster population aging than advanced countries in Europe and the United States [8], and drug-related problems have also increased. According to previous research, older people (aged 65 and over) took an average of 6.5–7.5 drugs, and the proportion of those who took 5 or more drugs was 44.1–67.4% [9,10]. The prevalence of potentially inappropriate medication (PIM) use based on the Beers Criteria was 70–80% [11,12], which was higher than that in the United States (39.9–56.6%) [13,14] and Europe (24.1–68.6%) [15,16,17,18].

The National Health Insurance (NHI) in Korea has health care delivery that relies heavily on private providers paid by fee-for-service, and there is no formal gate-keeping system resulting in competition among physician clinics and hospitals [19]. Patients can choose and visit the healthcare provider without limitations [20]. The physician is not available to check the medication prescribed by other physicians, except for checking the real-time pop-up alert provided when prescribing the few drugs listed on the drug utilization system [21]. This may be one reason for the challenges with comprehensive medication management for patients.

To reduce PIM use and manage the quality of medications, it is important to identify patient characteristics and prescriber factors for drug prescriptions that may result in PIM use. Polypharmacy, PIM use, and DDIs are iatrogenic factors related to healthcare providers [22,23], but few studies have considered prescriber-related factors. Previous studies have shown that continuity of care (COC) which is related to the reduction of drug-related problems [24,25]. Without a comprehensive medication management service in Korea, the relationship between COC and PIMs or DDIs needs to be investigated to improve long-term medication quality for older people.

This study investigated the prevalence of PIM use and DDIs in Korean older adults (aged 65 and over) using the 2015 Beers Criteria. To the best of our knowledge, this is the first study to identify the relationship between COC and prescriber factors and PIM use or DDIs in Korea. The patient- and prescriber-factors related to PIM use and DDIs were identified and analyzed to determine their associations with PIM use and DDIs.

## 2. Methods

### 2.1. Data and Study Population

South Korea has mandatory National Health Insurance (NHI), covering approximately 98% of the entire Korean population. The claims data of the Health Insurance Review and Assessment Service (HIRA) are collected when healthcare service providers submit a claim for reimbursement for a service that they provide to patients [26]. This study used data for representative sample, the HIRA—Adult Patient Sample (HIRA-APS), which is a random sample stratified by sex and age (classified as 5-year age groups) based on NHI claims data covering approximately 1 million patients aged ≥ 65 years (20%) [26]. The dataset contained the demographic characteristics, diagnoses, treatments, procedures, and prescriptions of patients. These data include all prescriptions for drugs covered by the NHI. We used the HIRA-APS data from 2016, and included only outpatient prescriptions during the study period. Patients who did not have at least one prescription within the fiscal year were excluded.

### 2.2. PIMs in Older Adults

PIM use and DDIs in this study were defined according to the 2015 Beers Criteria based on the list of medications and medication classes. The 2015 Beers Criteria added drug–drug interactions that should be avoided in older adults for the first time. The prevalence of PIM use and DDIs in 2016 was identified in older adults aged 65 years and above in Korea since the 2015 Beers Criteria have been updated. Among the medications listed as part of the 2015 Beers Criteria, those that were not licensed or distributed in Korea were excluded. Those that were not included in the Beers Criteria, but are approved and produced in Korea corresponding to the therapeutic class, were added. The target medications included in the analysis are presented in Supplementary Material (Table S1). However, insulin was excluded from the analysis because it is difficult to differentiate the sole use of short- or rapid-acting insulin from the titration of basal insulin in the claims data. The Beers Criteria recommend avoiding the sole use of short- or rapid-acting insulin to manage or prevent hyperglycemia in the absence of basal or long-acting insulin [7]. Medications targeted for PIM use were selected using the generic names on the Korean national reimbursed drug list.

### 2.3. Outcomes

We defined PIM use or DDIs on a per-patient basis as a patient who received at least one ambulatory care prescription associated with a PIM use or DDIs in 2016. The primary outcome was the proportion of patients who used prescriptions associated with PIM use or DDIs in patients who were prescribed at least one medication in 2016. The secondary outcomes were the number of prescriptions associated with PIM use or DDIs per older adult and the most prescribed drugs associated with PIM or DDIs. A frequency analysis was used to determine the most prescribed PIMs. The most common type of prescription associated with DDIs was also identified. We only defined DDI occurrence in individual prescriptions and could not address the possibility of interactions across different prescriptions. If there were multiple PIM medications or DDI types in the same prescription, it was counted as a separate case.

### 2.4. Covariates

Factors related to patients and prescriber were analyzed. The demographic factors included sex and age groups (65–69, 70–74, 75–79, and >80 years). The patients were stratified by health insurance type into Korean National Health Insurance (KNHI) subscribers and Medical Aid beneficiaries. Medical Aid is a medical assistance program for low-income people, and beneficiaries pay less in out-of-pocket payments than KNHI subscribers. The Charlson Comorbidity Index (CCI) was categorized as follows: 0, 1, 2, 3 or more. We also analyzed whether the patients had been hospitalized during the calendar year, and polypharmacy was defined as taking 5 or more medications based on previous studies [27,28,29]. Continuity of care (COC) is an important factor influencing the accessibility and continuity of healthcare services [24]. Generally, COC is measured by determining whether patients visited particular medical institutions or doctors. Older adults had a high COC if they made 75% or more of their total visits to the same doctor. As prescriber-related factors, the predominant medical center and the specialty of the physician from which patients received the largest number of prescriptions in the year were defined as the predominant medical center and specialty of the physician. Medical centers were classified as clinics, hospitals, secondary hospitals, and tertiary hospitals. The specialties were categorized into family medicine or internal medicine, surgery, neurology or psychiatry, and others.

### 2.5. Statistical Analysis

A descriptive analysis was performed of patients’ characteristics. To evaluate the differences between patients with and without PIM or DDI prescriptions, we used the chi-square test for categorical variables and the t-test for continuous variables. Multivariate logistic regression analysis was performed to analyze the characteristics of patients prescribed PIM or DDIs and prescriber characteristics. The odds ratios (ORs) for PIM use or DDIs and the corresponding 95% confidence intervals (CIs) were estimated, adjusting for possible covariates. Data management and analyses were conducted using SAS version 9.4 (SAS Institute, Inc., Cary, NC, USA).

## 3. Results

In 2016, 1,277,289 older adults received at least 1 outpatient prescription (Table 1). Female patients accounted for 58.0%, the average age of patients was 73.9 years (standard deviation, 7.0), and the age group with the highest number of patients was 65–69 years (33.0%). The proportion of patients who were prescribed 5 or more medications was 37.0%. A COC of over 0.75 was found for 8.9% of patients. Most patients (75.5%) predominantly visited clinics, and half of the patients (50.0%) visited family or internal medicine physicians. Of the older adults who received at least 1 prescription, 931,854 (73.0%) received 1 or more PIM prescriptions, and 169,871 (13.3%) received 1 or more DDI prescriptions.

**Figure 1:**
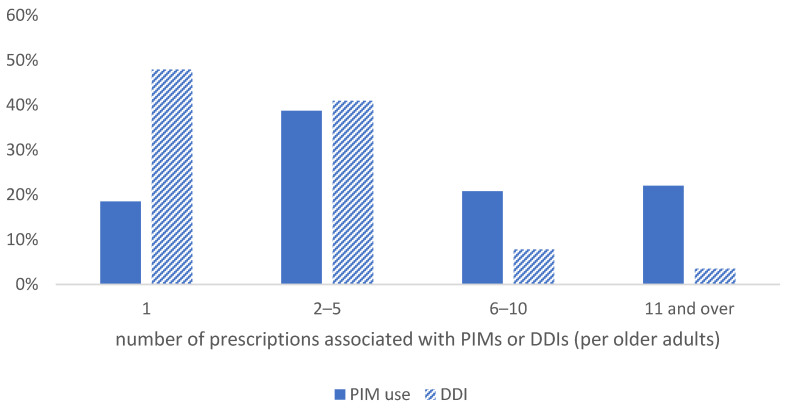
The number of PIM or DDI prescriptions per older adult. PIM, potentially inappropriate medication; DDI, drug–drug interaction.

Figure 1 shows the number of prescriptions associated with PIM use and DDIs among older adults who had at least 1 PIM or DDI prescription. Among the older adults who received at least 1 PIM prescription, it was most common to have 2–5 PIM prescriptions per year (38.7%), while 22.0% had 11 or more PIM prescriptions per year. Almost half of the older adults with DDI prescriptions (47.9%) received 1 DDI prescription, while 40.9% had 2–5 prescriptions.

The most common PIM ingredient included in the Beers Criteria was chlorphenamine, which was prescribed for 30.7% of the older adults with at least 1 PIM prescription and accounted for 12.7% of the prescriptions, followed by diazepam (20.4% of older adults with at least 1 PIM prescription, 11.5% of prescriptions). Anticholinergics, benzodiazepines, and non-steroidal anti-inflammatory drugs (NSAIDs) accounted for the majority of the top 15 drugs (Table 2).

Co-prescriptions of corticosteroids and NSAIDs accounted for 82.8% of DDIs among older adults with at least 1 DDI prescription and 77.3% of DDI prescriptions, followed by prescriptions of two more anticholinergics (15.8% of older adults with at least 1 DDI prescription and 15.2% of DDI prescriptions) (Table 3).

Table 4 presents predictors associated with PIM or DDI prescriptions. Women were more likely to have a PIM prescription than men (OR 1.22, 95% CI 1.21–1.23). As age increased, the likelihood of having a PIM prescription decreased (70–74 years: OR 1.08, 95% CI 1.07–1.09; 75–79 years: OR 1.07, 95% CI 1.05–1.08; 80 and over: OR 0.85, 95% CI 0.84–0.86; reference group: 65–69 years). Being a Medical Aid beneficiary having a higher CCI score and having been hospitalized were associated with a higher likelihood of PIM prescription. Polypharmacy was a critical factor, and patients with 5 or more prescription drugs had a higher likelihood of PIM prescription than patients receiving fewer than 5 prescriptions (OR 2.21, 95% CI 2.19–2.24). In addition, older adults who achieved high COC had a significantly lower likelihood of PIM prescription (OR 0.31, 95% CI 0.30–0.31). Regarding the prescriber-related factors, visiting a hospital as the predominant medical center decreased the likelihood of PIM prescriptions. Patients with the most prescriptions from surgeons or neurologists/psychiatrists also had a higher likelihood of PIM prescriptions.

The overall pattern for DDI prescriptions was similar. Female sex, being a Medical Aid beneficiary, having a higher CCI score, having been hospitalized, having polypharmacy, and mainly visiting surgeons or neurologists/psychiatrists were associated with a higher likelihood of DDI prescriptions. In contrast, older age, achieving a high COC, and mainly visiting a hospital were associated with a lower likelihood of DDIs.

## 4. Discussion

This study found that 73.0% of Korean older adults received PIM prescriptions at least once in 2016. Moreover, 13% of patients received prescriptions associated with DDIs more than once in 2016. This is consistent with the prevalence of PIM use of 70–81% measured using the 2012 Beers Criteria in South Korea [11,12] and 82.7% in Taiwan [30]. These results reflect a relatively high PIM prevalence compared to the prevalence in the U.S. or Europe. A partial explanation might relate to methodological differences between studies, and another possible explanation for the high PIM prevalence is not considering the duration of the prescription period or daily dose. However, a more important reason is the high prevalence of polypharmacy in Korean older adults. In the present study, 37.0% of older adults had polypharmacy, and older adults with polypharmacy had a higher prevalence of PIM use and DDIs. In other studies in Korea, the proportion of older adults with polypharmacy has been reported to be 44.1–86.4% [9,12,31,32] based on the methodological difference, which is relatively high compared to other countries (27% in Canada [33], 35.5% in the U.K. [34], 35.8% in Australia [35]). According to a systematic review of PIM predictors, polypharmacy was positively associated with PIM in all the included studies (27/27) [36]. The cultural background of preferring to take medicine partially explains the high prevalence of polypharmacy [37,38]. In addition, among older adults who had PIM prescriptions during the year, 20.8% had 6–10 prescriptions, and 22.0% had 11 or more prescriptions. Among older adults who received DDI prescriptions, 11.3% had more than 6 DDI prescriptions. This showed that some older adults were repeatedly exposed to prescriptions associated with PIM use or DDIs.

First-generation antihistamines and benzodiazepines were the most commonly prescribed PIM drugs. Most of the top 15 PIM drugs were those that act on the central nervous system such as antidepressants, benzodiazepines, and benzodiazepine receptor agonist hypnotics. Meanwhile, the majority of DDI prescriptions were co-prescriptions of corticosteroids and NSAIDs. This is consistent with the result of Yoon et al. (2018), who also reported that corticosteroid and NSAID co-prescriptions were the most common DDI prescriptions [39]. However, the study of Yoon et al. (2018) did not consider prescriber factors affecting occurrence of DDIs [39]. Among ambulatory care visits by patients aged ≥65 years in 2009 from the Taiwanese National Health Insurance Database, psychotropic drugs and first-generation antihistamines accounted for most of the top 10 PIMs [30]. Chen et al. reported that chlorphenamine was the second most-common PIM using Beers Criteria in four nursing home facilities in Malaysia [40]. A previous study reported that 23.5% of older patients received prescriptions of first-generation antihistamines, and these were more likely to be prescribed for treating common colds in Korea [41]. One possible explanation is that Korea’s national health insurance system makes it easy to visit medical institutions even with a cold and get a prescription. In addition, first-generation antihistamines are cheaper than second- or third-generation antihistamines based on the NHI reimbursement price. Thus, physicians may prefer to prescribe first-generation antihistamines. The panel experts agreed on including the prolonged use (>1 week) of first-generation antihistamines to the localized PIM list for Korean older adults based on the Delphi survey [42]. Meanwhile, the combination of NSAIDs and corticosteroids was not associated with old age, high risk of comorbidities, or depression, but was associated with skin disease [39]. The prescription of NSAIDs was frequent in older people (Table 2). The possible explanation is that additional corticosteroids may be prescribed for older people who are already taking NSAIDs.

The factors associated with an increased risk of PIM use or DDI were female sex, being a beneficiary of Medical Aid, a higher CCI, and polypharmacy, which was consistent with those of previous studies [13,15,30,43]. In contrast, a high COC had a significantly lower risk of PIM use or DDIs. These results are similar to those of previous studies on the number of prescribers of COC [44,45]. Regarding prescriber-related factors, patients using hospitals or general hospitals as primary medical institutions had a lower risk of PIM use than patients visiting clinics. These results reflect aspects of the Korean healthcare system. In Korea, there is no primary care physician system; instead, a fee-for-service system is in operation. Patients can visit multiple clinics at the same time, and clinic doctors cannot identify the medicines prescribed from other medical institutions unless they are drug-utilization-review drugs. Therefore, a plausible explanation for this finding is that this aspect of the medical system might make consistent patient management difficult and result in fragmentation of care, and contribute to a high prevalence of PIM use or DDI.

In South Korea, the risk of adverse drug reactions of long-acting benzodiazepines (e.g., diazepam) and antidepressants, which are widely prescribed for older adults, has been recognized, and systematic efforts have been made to reduce the use of these drugs. Since October 2015, a real-time drug-utilization-review program has been implemented for long-acting benzodiazepines and tricyclic antidepressants among drugs prescribed to patients aged 65 years and above. A pop-up window opens at the time of the prescription to inform the prescriber about adverse drug reactions and for the prescriber to enter the reason for the prescription [46]. Following an interrupted time series analysis, a nationwide prospective DUR implementation immediately lowered the prevalence of older adults prescribed drugs associated with PIM by 0.49% (95% CI −0.60, −0.37) [46].

However, an alert system, such as DUR, was effective in reducing the incidence of new prescriptions associated with PIM use, but it had no significant effect on the discontinuation of already prescribed drugs associated with PIM use [47]. Recently, pharmacist-led medication review services have become available in several countries such as the United Kingdom (Medicines Use Review), the United States (Medication Therapy Management), Australia (Home Medication Review), and Canada (MedsCheck) [48]. Positive results were found for reducing the mean number of medicines prescribed, those associated with PIM use, and hospitalizations [49,50,51,52,53]. Therefore, considering implementing these pharmacist-led medication review in the healthcare system is highly encouraged and this system will aid to reduce PIMs and DDIs in older adults who are benefited from the high accessibility to the health care system in Korea.

This study has some limitations. First, only prescription drugs for ambulatory care were included in the analysis. Medication decisions can differ for inpatient and ambulatory care visits; we only included ambulatory care prescriptions. Since drugs for self-medication, such as OTC medicines, purchased and taken by individuals, were not considered because this study used cross-sectional claims data, there is a possibility that PIM use was underestimated. Second, no data on the reasons for the prescriptions were available. It is reasonable for a physician to prescribe a PIM if the benefit of the medication would be greater than the risk. Regarding DDIs, it was not confirmed whether drugs for gastrointestinal protection were prescribed together to prevent peptic ulcer or gastrointestinal bleeding caused by the combination of corticosteroids and NSAIDs. Third, drug-disease interactions were excluded from the analysis because the clinical status of the patient could not be clearly determined based on the cross-sectional data. Finally, adverse drug reactions and health outcomes caused by PIM use could not be estimated. Therefore, further studies are needed to confirm the effects of PIM use on health outcomes.

Nevertheless, the strengths of this study are as follows. First, since claims data were used, the possibility of recall bias for prescription drug use and medical use was relatively small. Second, the recent information on DDIs added in 2015 was evaluated. In detail, it was confirmed that the majority of inappropriate co-prescriptions were for corticosteroid use with NSAIDs. This shows that DDI reduction can be expected by improving awareness through education on the interaction between these two drug types. In particular, training for osteoarthritis patients and surgeons can be expected to reduce the incidence of DDIs, because the risk is relatively high in patients with osteoarthritis and in patients who undergo surgery.

## 5. Conclusions

This study confirmed that a significant number of adults aged 65 and over in Korea received PIM prescriptions, and more than half of the patients received multiple PIM prescriptions during the year. Most of the DDI prescriptions were NSAIDs and corticosteroids, showing that the combination of these two drug types was quite frequent. In addition, PIM or DDI prescriptions were higher in patients who had a low COC or mainly visited clinics; therefore, patients with these characteristics need to be considered as the preferred intervention targets to reduce prescriptions associated with PIM use and DDIs. There is a need for a medication management program suitable for the Korean system to facilitate more comprehensive and safer use of medicines for older adults.

## Figures and Tables

**Table 1:** Characteristics of older adults and prevalence of PIM use or drug–drug interactions.

		Older Adults	PIM Use	DDI
		*N* (%)	*N* (%)	*N* (%)
**Total**		1,277,289 (100)	931,854 (100)	169,871 (100)
Sex	Male	536,592 (42.0)	377,552 (40.5)	66,840 (39.3)
	Female	740,697 (58.0)	554,302 (59.5)	103,031 (60.7)
Age	Mean (SD)	73.9 (7.0)	73.7 (6.4)	73.2 (6.1)
	65–69	421,973 (33.0)	300,444 (32.2)	57,848 (34.1)
	70–74	343,011 (26.9)	255,779 (27.4)	48,650 (28.6)
	75–79	264,407 (20.7)	199,800 (21.4)	36,278 (21.4)
	≥80	247,898 (19.4)	175,831 (18.9)	27,095 (16)
Type of insurance	NHI	1184,926 (92.8)	860,857 (92.4)	156,885 (92.4)
	Medical Aid	92,363 (7.2)	70,997 (7.6)	12,986 (7.6)
CCI	0	676,024 (52.9)	468,870 (50.3)	84,008 (49.5)
	1	343,081 (26.9)	261,696 (28.1)	49,592 (29.2)
	2	162,332 (12.7)	124,472 (13.4)	22,372 (13.2)
	≥3	95,852 (7.5)	76,816 (8.2)	13,899 (8.2)
Hospitalization	No	936,655 (73.3)	663,459 (71.2)	116,666 (68.7)
	Yes	340,634 (26.7)	268,395 (28.8)	53,205 (31.3)
Polypharmacy	No	804,520 (63.0)	543,479 (58.3)	92,905 (54.7)
	Yes	472,769 (37.0)	388,375 (41.7)	76,966 (45.3)
COC	<0.75	1,163,141 (91.1)	876,691 (94.1)	163,782 (96.4)
	≥0.75	114,148 (8.9)	55,163 (5.9)	6089 (3.6)
Type of predominant medical center	Clinic	964,362 (75.5)	716,200 (76.9)	138,899 (81.8)
	Hospital	83,004 (6.5)	58,623 (6.3)	9468 (5.6)
	Secondary hospital	151,237 (11.8)	106,473 (11.4)	14,432 (8.5)
	Tertiary hospital	78,686 (6.2)	50,558 (5.4)	7072 (4.2)
Specialty of the physician ^1^	Family or internal medicine	638,658 (50.0)	462,988 (49.7)	81,079 (47.7)
	Surgery	224,730 (17.6)	170,128 (18.3)	34,701 (20.4)
	Neurology or psychiatry	63,716 (5.0)	48,041 (5.2)	6819 (4.0)
	Others	350,185 (27.4)	250,697 (26.9)	47,272 (27.8)

^1^ Specialty of the physician refers to the specialty of the physician who prescribed the most prescriptions. PIM, potentially inappropriate medication; DDI, drug–drug interaction; SD, standard deviation; NHI, National Health Insurance; CCI Charlson comorbidity index; COC, continuity of care.

**Table 2:** Top 15 prescribed drugs in potentially inappropriate medications.

PIMs per Older Adult			PIMs per Prescription		
Drug	No. of Older Adults (*N* = 931,854)	%	Drug	No. of Prescriptions (*N* = 6,554,063)	%
First-generation antihistamine	536,334	57.6	First-generation antihistamine	1,674,582	25.6
Chlorphenamine	286,475	30.7	Chlorphenamine	830,122	12.7
Dimenhydrinate	143,161	15.4	Dimenhydrinate	527,907	8.1
Hydroxyzine	106,698	11.5	Hydroxyzine	316,553	4.8
NSAID	425,176	45.6	Benzodiazepines	1,605,629	24.5
Meloxicam	127,995	13.7	Diazepam	752,516	11.5
Mefenamic acid	104,184	11.2	Alprazolam	600,614	9.2
Ibuprofen	100,138	10.7	Etizolam	252,499	3.9
Naproxen	92,859	10.0	NSAID	1,156,500	17.6
Benzodiazepines	385,886	41.4	Meloxicam	503,487	7.7
Diazepam	190,461	20.4	Mefenamic acid	243,344	3.7
Alprazolam	132,022	14.2	Ibuprofen	228,311	3.5
Etizolam	63,403	6.8	Naproxen	181,358	2.8
Proton-pump inhibitor	259,840	27.9	Proton-pump inhibitor	715,006	10.9
Rabeprazole	157,563	16.9	Rabeprazole	526,049	8.0
Lansoprazole	65,999	7.1	Lansoprazole	188,957	2.9
Omeprazole	36,278	3.9			
Nonbenzodiazepine, benzodiazepine receptor agonist hypnotics			Nonbenzodiazepine, benzodiazepine receptor agonist hypnotics		
Zolpidem	102,630	11.0	Zolpidem	447,641	6.8
Antidepressants			Antidepressants		
Amitriptyline TD	50,602	5.4	Amitriptyline TD	224,246	3.4
			Peripheral alpha-1 blockers		
			Terazosin	155,386	2.4

PIM, potentially inappropriate medication.

**Table 3:** Top 5 prescribed drug–drug interactions.

Type of Drug–Drug Interactions		Per Older Adult		Per Prescription	
		No. of Older Adults (*N* = 169,871)	%	No. of Prescriptions (*N* = 475,577)	%
Corticosteroids	NSAIDs	140,627	82.8	367,370	77.3
Anticholinergic	Anticholinergic	26,812	15.8	72,265	15.2
Antidepressants, antipsychotics, and benzodiazepines *		3882	2.3	20,009	4.2
Theophylline	Cimetidine	2272	1.3	7488	1.6
Peripheral alpha-1 blockers	Loop diuretics	1316	0.8	5854	1.2

* Benzodiazepines and nonbenzodiazepine, benzodiazepine receptor agonist hypnotics; DDI, drug–drug interaction; NSAIDs, non-steroidal anti-inflammation drugs.

**Table 4:** Predictors of PIM use and drug–drug interactions among older adults.

Variables		PIM Use			DDIs		
		OR	95% CI		OR	95% CI	
Sex	Male (Ref)						
	Female	1.22	1.21	1.23	1.30	1.28	1.31
Age	65–69 (ref)						
	70–74	1.08	1.07	1.09	1.05	1.03	1.06
	75–79	1.07	1.05	1.08	0.97	0.96	0.99
	80 and over	0.85	0.84	0.86	0.65	0.64	0.67
Type of insurance	NHI (ref)						
	Medical Aid	1.12	1.10	1.14	1.14	1.11	1.17
CCI	0 (ref)						
	1	1.25	1.24	1.26	1.35	1.33	1.37
	2	1.20	1.18	1.21	1.18	1.15	1.20
	3 and over	1.33	1.30	1.35	1.31	1.27	1.34
Hospitalization	No (ref)						
	Yes	1.47	1.45	1.48	1.74	1.72	1.77
Polypharmacy	No (ref)						
	Yes	2.21	2.19	2.24	2.73	2.69	2.76
COC	<0.75 (ref)						
	≥0.75	0.31	0.30	0.31	0.20	0.19	0.20
Type of predominant medical center	Clinic (ref)						
	Hospital	0.76	0.75	0.77	0.61	0.60	0.63
	Secondary hospital	0.66	0.65	0.67	0.44	0.43	0.45
	Tertiary hospital	0.48	0.47	0.49	0.32	0.31	0.33
Specialty of the physician *	Family or internal medicine (ref)						
	Surgery	1.20	1.18	1.21	1.42	1.40	1.45
	Neurology or psychiatry	1.21	1.18	1.23	1.07	1.03	1.10
	Others	1.01	1.00	1.02	1.10	1.09	1.12

* Specialty of the physician refers to the specialty of the physician who prescribed the most prescriptions.; PIM, potentially inappropriate medication; DDI, drug–drug interaction; NHI, National Health Insurance; CCI, Charlson comorbidity index; COC, continuity of care.

## Data Availability

This study used National Health Insurance Service claims data. These third-party data were obtained from the Korea Health Insurance Review and Assessment Service (HIRA). The authors had no special access privileges to the data. Interested researchers can apply and purchase access to the data by contacting the Healthcare Bigdata Hub (https://opendata.hira.or.kr/home.do).

## Supplementary Materials

The following are available online at https://www.mdpi.com/article/10.3390/jcm10112305/s1, Table S1. Beers Criteria for Potentially Inappropriate Medication Use in Older Adults, Modified According to the Korean Context.
