# Supplementary material for: Patient- and Prescriber-Related Factors Associated with Potentially Inappropriate Medications and Drug–Drug Interactions in Older Adults
Source: J Clin Med. 2021 May 25;10(11):2305. doi: 10.3390/jcm10112305 (PMC8198936; doi:10.3390/jcm10112305)
Supplement: Supplementary file 1 [file jcm-10-02305-s001.zip]

Supplemental Material

**Table 1.** Beers Criteria for Potentially Inappropriate Medication Use in Older Adults, Modified According to the Korean Context.

| **Organ System** | **Therapeutic Category** | **Drugs** |
| --- | --- | --- |
| **Anticholinergics** | First-generation antihistamines | Brompheniramine |
|  |  | Carbinoxamine |
|  |  | Chlorpheniramine |
|  |  | Clemastine |
|  |  | Cyproheptadine |
|  |  | Dexbrompheniramine |
|  |  | Dexchlorpheniramine |
|  |  | Dimenhydrinate |
|  |  | Diphenhydramine (oral) |
|  |  | Doxylamine |
|  |  | Hydroxyzine |
|  |  | Meclizine |
|  |  | Promethazine |
|  |  | ebastine |
|  |  | oxatomide |
|  |  | Triprolidine |
|  | Antiparkinsonian agents | Benztropine (oral) |
|  |  | Trihexyphenidyl |
|  | Antispasmodics | Atropine (excludes ophthalmic) |
|  |  | Belladonna alkaloids |
|  |  | Clidinium-Chlordiazepoxide |
|  |  | Dicyclomine |
|  |  | Hyoscyamine |
|  |  | Propantheline |
|  |  | Scopolamine (hyoscine) |
| **Antithrombotics** | Dipyridamole, oral short-acting (does not apply to the extended-release combination with aspirin) | Dipyridamole |
|  | Ticlopidine | Ticlopidine |
| **Anti-infective** | Nitrofurantoin | Nitrofurantoin |
| **Cardiovascular** | Peripheral alpha-1 blockers | Doxazosin |
|  |  | Prazosin |
|  |  | Terazosin |
|  | Central alpha blockers | Clonidine |
|  |  | Guanabenz |
|  |  | Guanfacine |
|  |  | Methyldopa |
|  |  | Reserpine (>0.1 mg/d) |
|  | Disopyramide | Disopyramide |
|  | Dronedarone | Dronedarone |
|  | Digoxin | Digoxin |
|  | Nifedipine, immediate release | Nifedipine, immediate release |
|  | Amiodarone | Amiodarone |
| **Central nervous system** | Antidepressants, alone or in combination | Amitriptyline |
|  |  | Amoxapine |
|  |  | Clomipramine |
|  |  | Desipramine |
|  |  | Doxepin >6 mg/d |
|  |  | Imipramine |
|  |  | Nortriptyline |
|  |  | Paroxetine |
|  |  | Protriptyline |
|  |  | Trimipramine |
|  | Antipsychotics, first- (conventional) and second- (atypical) generation | prochlorperazine |
|  | (1st generation) | chlorpromazine |
|  |  | promazine |
|  |  | triflupromazine |
|  |  | levomepromazine |
|  |  | fluphenazine |
|  |  | trifluoperazine |
|  |  | perphenazine |
|  |  | thioridazine |
|  |  | mesoridazine |
|  |  | pericyazine |
|  |  | pipotiazine |
|  |  | pimozide |
|  |  | fluspirilene |
|  |  | molindone |
|  |  | clotiapine |
|  |  | loxapine |
|  |  | haloperidol |
|  |  | bromperidol |
|  |  | benperidol |
|  |  | droperidol |
|  |  | thiothixene |
|  |  | clopenthixol |
|  |  | zuclopenthixol |
|  |  | fluphenthixol |
|  |  | olanzapine |
|  | (2nd generation) | clozapine |
|  |  | brexipiprazole |
|  |  | aripiprazole |
|  |  | asenapine |
|  |  | quetiapine |
|  |  | lurasidone |
|  |  | paliperidone |
|  |  | risperidone |
|  |  | iloperidone |
|  |  | ziprasidone |
|  |  | pimavanserin |
|  | Barbiturates | Amobarbital |
|  |  | Butabarbital |
|  |  | Butalbital |
|  |  | Mephobarbital |
|  |  | Pentobarbital |
|  |  | Phenobarbital |
|  |  | Secobarbital |
|  | Benzodiazepines (short, intermediate) | Alprazolam |
|  |  | Estazolam |
|  |  | Lorazepam |
|  |  | Oxazepam |
|  |  | Temazepam |
|  |  | Triazolam |
|  |  | etizolam |
|  |  | Midazolam |
|  |  | brotizolam |
|  |  | clotiazepam |
|  | Benzodiazepines (long) | Clorazepate |
|  |  | Chlordiazepoxide (alone or in combination with amitriptyline or clidinium) |
|  |  | Clonazepam |
|  |  | Diazepam |
|  |  | Flurazepam |
|  |  | bromazepam |
|  |  | flunitrazepam |
|  |  | Flutoprazepam |
|  |  | Pinazepam |
|  |  | Nordazepam |
|  |  | Quazepam |
|  | Nonbenzodiazepine, benzodiazepine receptor agonist hypnotics | Eszopiclone |
|  |  | Zolpidem |
|  |  | Zaleplon |
|  | Ergoloid mesylates (dehydrogenated ergot alkaloids) | Ergoloid mesylates (dehydrogenated ergot alkaloids) |
|  | Isoxsuprine | Isoxsuprine |
| **Endocrine** | Androgens | Methyltestosterone |
|  |  | Testosterone |
|  | Estrogens with or without progestins | Estrogens with or without progestins |
|  | Megestrol | Megestrol |
|  | Sulfonylureas, long-duration | Chlorpropamide |
|  |  | Glyburide (Glibenclamide) |
| **Gastrointestinal** | Metoclopramide | Metoclopramide |
|  | Proton-pump inhibitors | omeprazole |
|  |  | esomeprazole |
|  |  | lansoprazole |
|  |  | dexlansoprazole |
|  |  | rabeprazole |
|  |  | pantoprazole |
|  |  | ilaprazole |
| **Pain medications** | Meperidine | Meperidine(pethidine) |
|  | NSAID | Aspirin >325 mg/d Diclofenac |
|  |  | Diflunisal |
|  |  | Etodolac |
|  |  | Fenoprofen |
|  |  | Ibuprofen |
|  |  | Ketoprofen |
|  |  | Meclofenamate |
|  |  | Mefenamic acid |
|  |  | Meloxicam |
|  |  | Nabumetone |
|  |  | Naproxen |
|  |  | Oxaprozin |
|  |  | Piroxicam |
|  |  | Sulindac |
|  |  | Tolmetin |
|  |  | Indomethacin |
|  |  | Ketorolac, includes parenteral |
|  | Pentazocine | Pentazocine |
|  | Skeletal muscle relaxants | Carisoprodol |
|  |  | Chlorzoxazone |
|  |  | Cyclobenzaprine |
|  |  | Metaxalone |
|  |  | Methocarbamol |
|  |  | Orphenadrine |
|  | Genitourinary | Desmopressin |
